# Evaluating research co-production: protocol for the Research Quality Plus for Co-Production (RQ+ 4 Co-Pro) framework

**DOI:** 10.1186/s43058-022-00265-7

**Published:** 2022-03-14

**Authors:** Robert K. D. McLean, Fred Carden, Ian D. Graham, Alice B. Aiken, Rebecca Armstrong, Judy Bray, Christine E. Cassidy, Olivia Daub, Erica Di Ruggiero, Leslie A. Fierro, Michelle Gagnon, Alison M. Hutchinson, Roman Kislov, Anita Kothari, Sara Kreindler, Chris McCutcheon, Jessica Reszel, Gayle Scarrow

**Affiliations:** 1 Policy and Evaluation Division, International Development Research Centre, Ottawa, Canada; 2 Integrated Knowledge Translation Research Network, Ottawa Hospital Research Institute, Ottawa, Canada; 3 Using Evidence Inc., Ottawa, Canada; 4 Clinical Epidemiology Program, Ottawa Hospital Research Institute & Schools of Epidemiology and Public Health & Nursing, University of Ottawa, Ottawa, Canada; 5 Dalhousie University, Halifax, Canada; 6 Research and Evaluation Branch, National Disability Insurance Agency, Melbourne, Australia; 7 Canadian Cancer Society, Toronto, Canada; 8 School of Nursing, Dalhousie University, Halifax, Canada; 9 Dalla Lana School of Public Health, University of Toronto, Toronto, Canada; 10 Max Bell School of Public Policy, McGill University, Montreal, Canada; 11 Consultant, Ottawa, Canada; 12 School of Nursing and Midwifery, Centre for Quality and Patient Safety in the Institute for Health Transformation, Deakin University, Geelong, Australia & Monash Health, Melbourne, Australia; 13 Faculty of Business and Law, Manchester Metropolitan University, Manchester, UK & School of Health Sciences, The University of Manchester, Manchester, UK; 14 School of Health Studies, Western University, London, Canada; 15 Department of Community Health Sciences, University of Manitoba, Winnipeg, Canada; 16 George & Fay Yee Centre for Healthcare Innovation, University of Manitoba, Winnipeg, Canada; 17 Ottawa Hospital Research Institute, Ottawa, Canada & School of Nursing, University of Ottawa, Ottawa, Canada; 18 Michael Smith Health Research B.C., Vancouver, Canada

## Abstract

**Background:**

Research co-production is an umbrella term used to describe research users and researchers working together to generate knowledge. Research co-production is used to create knowledge that is relevant to current challenges and to increase uptake of that knowledge into practice, programs, products, and/or policy. Yet, rigorous theories and methods to assess the quality of co-production are limited. Here we describe a framework for assessing the quality of research co-production—Research Quality Plus for Co-Production (RQ+ 4 Co-Pro)—and outline our field test of this approach.

**Methods:**

Using a co-production approach, we aim to field test the relevance and utility of the RQ+ 4 Co-Pro framework. To do so, we will recruit participants who have led research co-production projects from the international Integrated Knowledge Translation Research Network. We aim to sample 16 to 20 co-production project leads, assign these participants to dyadic groups (8 to 10 dyads), train each participant in the RQ+ 4 Co-Pro framework using deliberative workshops and oversee a simulation assessment exercise using RQ+ 4 Co-Pro within dyadic groups. To study this experience, we use a qualitative design to collect participant demographic information and project demographic information and will use in-depth semi-structured interviews to collect data related to the experience each participant has using the RQ+ 4 Co-Pro framework.

**Discussion:**

This study will yield knowledge about a new way to assess research co-production. Specifically, it will address the relevance and utility of using RQ+ 4 Co-Pro, a framework that includes context as an inseparable component of research, identifies dimensions of quality matched to the aims of co-production, and applies a systematic and transferable evaluative method for reaching conclusions. This is a needed area of innovation for research co-production to reach its full potential. The findings may benefit co-producers interested in understanding the quality of their work, but also other stewards of research co-production. Accordingly, we undertake this study as a co-production team representing multiple perspectives from across the research enterprise, such as funders, journal editors, university administrators, and government and health organization leaders.

**Supplementary Information:**

The online version contains supplementary material available at 10.1186/s43058-022-00265-7.

Contributions to the literature

- Research co-production is a strategy used to produce scientific and societal benefits. Co-production is used to create research that is more relevant to knowledge users and to increase uptake of research into practice, programs, products, and policy. Yet, rigorous theories and methods to assess the quality of co-production are limited.
- Adapted from the validated Research Quality Plus (RQ+) approach, Research Quality Plus for Co-Production Research (RQ+ 4 Co-Pro) presents an adaptable and modular framework for evaluating co-production quality that addresses the context of the research, its scientific rigor, its legitimacy, and how well it is positioned for use.
- This study is the first RQ+ 4 Co-Pro application. It will field test the utility of the framework for evaluating research co-production projects, using a sample of completed projects from an international research network: the Integrated Knowledge Translation Research Network (IKTRN).
- A field-tested co-production-specific evaluation approach will contribute to the critical development of high-quality research co-production as a means of knowledge generation and application.

## Background

Research co-production shows great promise for connecting science to societal problems. Research co-production can be rigorous and ethical [1–7] and serve as a vehicle for generating and translating scientific findings into action [8]. Research on implementation science and scaling science [9] demonstrates that the use of rigorous research designs is only one consideration when implementing and scaling innovations—context, user/beneficiary perspectives, and systems matter just as much. The active involvement of users (those who may move research findings in action) and beneficiaries (those who may be affected) can be a crucial predictor of success [8–11].

Research co-production comes in many forms and under many different names. Among others, community academic partnership [11]; community-based participatory research [12]; co-creation [13]; and integrated knowledge translation [14–16]. A research study involving experts from a range of five research co-production traditions [17] found that the definitions and motivations of each type of co-production research were very similar. While there are many different names, engaging the users of research in the research process is a common goal. Therefore, we anticipate the results of this study to hold potential beyond the immediate sample. See Table 1 for general definitions of selected co-production traditions.

**Table 1:** Research co-production traditions

Tradition	Definition
Participatory Research	In participatory research the community is part of shaping the research agenda; community members work with researchers on the research itself and on implementation of the agenda [18].
Integrated Knowledge Translation	As defined on the Integrated Knowledge Translation Research Network website, ‘Integrated Knowledge Translation (IKT) is a model of collaborative research, where researchers work with knowledge users who identify a problem and are in a position to act on the research findings [19].’ In short, it is about doing research with the people who use it.
Engaged Scholarship	Similar to the traditions above, Engaged Scholarship engages communities with researchers at multiple stages of the research process and focuses on issues that are important to a community. A community may be geographic or a community of interest (e.g., patient engagement in research that affects them) [20].
Mode 2 Research	Mode 2 research is a transdisciplinary approach to research on development problems that engages both researchers and practitioners without strict hierarchy or fixed approaches in the research. The research is co-produced with people who work and live in the domain of the research [21, 22].
Community Academic Partnership	Community-academic partnerships optimize the engagement of academic and community resources thereby increasing the pertinence of academic research and trust in findings in the community. Community-academic partnerships support diverse solutions to meet the needs of specific communities [23].
Research Co-Production	Research co-production is an umbrella term. The term is used to describe the process of researchers working with research users to create and conduct research together. The aim of research co-production is to bring multiple perspectives into setting research questions and into decision-making about the how the research is done, so that the work reflects the needs of those who will use it [24].

### The need for better evaluation approaches for co-production

There is growing dissatisfaction with the approaches available for assessing the quality of research co-production. Traditional approaches to research quality assessment do not take into account engagement with knowledge users and, as such, do not address a key factor in the hypothesis behind research co-production: that meaningful researcher - knowledge user partnerships make a difference to the quality of the evidence that research produces [8, 10].

Table 2 outlines the predominant forms of research evaluation—as classified and further discussed in Chapter 4.3 of Research Coproduction in Healthcare [25]—and describes how these approaches can undervalue research co-production.

**Table 2:** Mainstream evaluation stacked against co-production [25]

Evaluation approach	Challenges for co-production
**Deliberative** *What form does it take?* Peer-review at proposal, ethics, publication, and sharing stages of research.	Peer-review relies on researchers, not users or beneficiaries, to judge a proposal or a project in terms of scientific criteria. With few exceptions co-production proposals are assessed by scientific peers, not knowledge users (who are not considered peers). (See for example the work of PCORI (www.PCORI.org) or the former Knowledge Translation Funding Program at CIHR [8, 26] for examples of ‘Merit Review’ in practice.) Further, they use scientific criteria and scientist perspectives to determine whether, 1) a study is ethical for participants on behalf of participants (through REB procedures), and, 2) if a study contains publishable results, not actionable results. In our view, scientists’ expertise can identify the knowledge gaps the work aims to fill and critique the strength of the methods that will be used to produce it. Yet, without including knowledge users and beneficiaries’ significant evaluation gaps persist, as knowledge users are best placed to assess the relevance, significance, utility, and potential impact of the research.
**Analytic** *What form does it take?* Metrics and quantitative indices. For example, bibliometrics, altmetrics, university rankings, journal rankings*.*	Metrics are biased toward fields of research where productivity in creating output is paramount, largely, the scholarly paper published in a peer-reviewed, indexed journal. They are also biased toward the quantification of outputs. Metrics and their aggregations tell us little, if anything, about the quality of the engagement of users in a project. Neither do they speak to the policy or practice relevance of a research topic, or the actual implications of the work for intended beneficiaries. Moreover, they are largely blind to research results that fall outside the indices of mainstream, English-language, academic journal publishing. Similarly, real-world impact resulting from co-production typically goes uncounted with the analytic paradigm.
**Research impact assessment (RIA)** *What form does it take?* Retrospective reviews, often case studies with social and economic measures.	For co-producers whose aim is knowledge uptake and use, the RIA approach seems welcome at first glance. In some cases, the RIA may even privilege research co-production which can be well positioned to accelerate the uptake and impact of research by knowledge users. However, RIA is not a complete solution for research co-production quality evaluation. RIA may provide a meaningful measure for funders and organizations whose primary concern is amplifying or modifying the magnitude of impact they can demonstrate and communicate; additionally, it does not systematically recognize and study the process of user-engagement and how it can set a course and even create social change *during* study design and implementation [27, 28]. Furthermore, the mismatch between research funding trajectories (typically 1-5 years) and research impact trajectories (typically 10-20 years) leaves a significant gap in our knowledge of how to do better co-production.

### Objectives of the RQ+ 4 Co-Pro field test

**Fig. 1:**
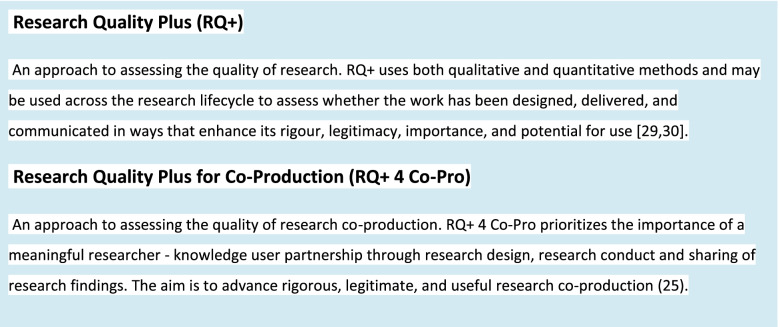
Key definitions

The purpose of this study is to field test the relevance and utility of an adapted research quality evaluation approach that was first developed and validated by the International Development Research Centre. This approach, called Research Quality Plus (RQ+) [29, 30], has previously been used to assess applied and use-oriented research. For a full explication, see McLean et al. [29]. With this study, we will test whether RQ+ can be adapted for assessing the quality of co-production research^1^. This prototype adaptation is called the Research Quality Plus for Co-Production, or RQ+ 4 Co-Pro, framework (25). See Fig. 1 below for key definitions of RQ+ and RQ+ 4 Co-Pro.

Two research questions guide the field test:

1. Is the RQ+ 4 Co-Pro framework relevant for the evaluation of research co-production?
2. Is the RQ+ 4 Co-Pro framework useful for the evaluation of research co-production?

## The Research Quality Plus for Co-Production (RQ+ 4 Co-Pro) framework

**Fig. 2:**
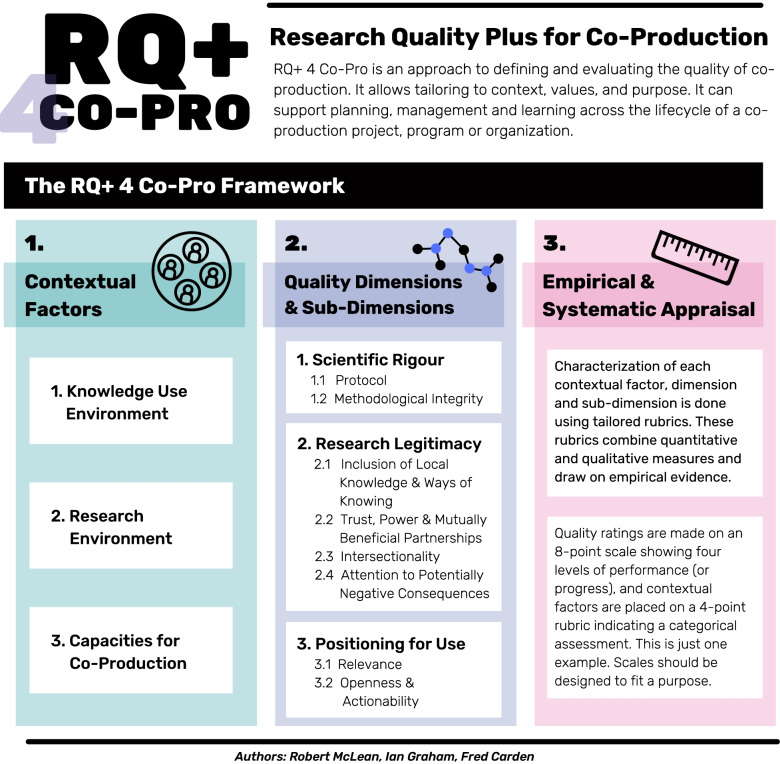
The RQ+ 4 Co-Pro framework [adapted from infographic originally published by authors (RKDM, IDG, FC), and secondly in 25]

The adaptation of RQ+ into the RQ+ 4 Co-Pro framework is illustrated in Fig. 2. RQ+ 4 Co-Pro was first proposed by authors of this paper following their experience designing and using the initial RQ+ framework at IDRC, doing research evaluations internationally, and doing research co-production (25). This is a prototype rendition. The study described in this manuscript aims to field test the protype. In Additional file 1: Appendix I, the fully detailed RQ+ 4 Co-Pro framework template is provided for the interested reader; it includes the definitions of each framework component and the associated evaluative rubrics. Additional file 2: Appendix II provides a crosswalk of the components of the RQ+ framework with the components of the RQ+ 4 Co-Pro framework.

The RQ+ 4 Co-Pro framework embraces the three tenets of the RQ+ Approach. These are as follows: (1) context matters, (2) quality is multi-dimensional, and (3) assessments should be empirical and systematic. These are modified from the RQ+ framework to reflect the particularities of co-production research. Here we provide a description of how each tenet was tailored, and then introduce an all-of-framework infographic (Fig. 2) to show how the three tenets fit together.

### Contextual factors

Research always occurs in a context. Research is affected by and affects the socio-economic, historical, cultural, and political contexts as well as the geographic and institutional setting.^2^ Attention to context is particularly important in the evaluation of the quality of research co-production [31]. We identify three contextual factors that can be monitored and categorized in a co-production evaluation. The goal in examining contextual factors is to gather information that can help to understand and navigate the enabling environment for co-production research. Understanding context is important to research design, management, and funding decisions as it helps clarify potential risks and opportunities and might also help with the development of strategies to capitalize on these and monitor progress. The contextual factors are not intended to affect the ratings of research quality dimensions or sub-dimensions, nor is any rating of a contextual factor necessarily “better” than another. Rather, they help to provide a deeper understanding of the enabling environment.

The three RQ+ 4 Co-Pro contextual factors are as follows: (1) Knowledge Use Environment, (2) Research Environment, and (3) Capacities for Co-Production. In the International Development Research Centre’s current RQ+ framework, there are five contextual factors. Three are closely aligned to those here, given some tailoring to match co-production specifically. The additional two contextual factors, Data Environment and Maturity of the Research Field, are not included in RQ+ 4 Co-Pro as they present less immediate alignment with the aims of co-production. The decision to reduce the number and tailor the contextual factors for RQ+ 4 Co-Pro was the result of consultations between authors of this paper, and their shared experiences with co-production and co-production evaluations [25]. With this field test, we will further examine the relevance of these three contextual factors and determine the need to modify, exclude, or include new elements on grounds of relevance and/or utility (see research questions above). Additional file 2: Appendix II provides a crosswalk of the RQ+ contextual factors vis-à-vis the RQ+ 4 Co-Pro contextual factors.

### Quality dimensions and sub-dimensions

To assess co-production quality, we identify three dimensions and eight sub-dimensions. These are summarized in Fig. 2 and presented in detail in Additional file 1: Appendix I. Additional file 2: Appendix II crosswalks these dimensions and sub-dimensions with those of the RQ+ framework.

As with all research, *Scientific Rigour* is central to co-production research and therefore comprises the first dimension. Two sub-dimensions are identified under *Scientific Rigour*: 1.1. Protocol which addresses issues of study design, and 1.2. Methodological Integrity which assesses the rigor and integrity of the application of the study design. *Research Legitimacy* is the second dimension of RQ + 4 Co-Pro. There are four sub-dimensions to *Research Legitimacy* that assess the fidelity of the research to the operating environment. These are as follows: 2.1 Inclusion of Local Knowledge and Ways of Knowing, 2.2. Trust, Power and Mutually Beneficial Partnerships, 2.3. Intersectionality, and 2.4. Attention to Potentially Negative Consequences. The third and final dimension is *Positioning for Use*. It assesses the utility of the co-production research through examining 3.1. Relevance or how well the work is aligned to a current problem, and 3.2. Openness and Actionability which addresses accessibility and usefulness of the research findings.

In the RQ+ 4 Co-Pro framework, all dimensions are interrelated and should not be considered as variables that are independent of each other. They are disaggregated to promote a deeper understanding of the multiple dimensions of research quality—ultimately, they must be considered as a set. We assign equal weight to the dimensions and sub-dimensions; others may choose to prioritize or highlight some sub-dimensions over others in any assessment they design.

### Empirical and systematic appraisal

Column 3 in Fig. 2 outlines the scale to be used for measurement. RQ+ 4 Co-Pro users apply a rubric for measurement which ensures transparency in the results and promotes a systematic approach across all the research that is being assessed. A combination of qualitative explanations and quantitative measures of sub-dimensions should be used to reach conclusions about the quality of the co-production research. In the following sections, we outline how empirical evidence will be gathered in our field test.

Table 3 below outlines how RQ+ 4 Co-Pro addresses some key recommendations from studies on research co-production approaches.

**Table 3:** What *RQ+ 4 Co-Pro* can learn, benefit from, and build on from existing frameworks, experiences, and systematic reviews [25]

Article/framework and theoretical lens	Recommendation (from the paper cited)	Lesson for ***RQ+ 4 Co-Pro***
*Integrated Knowledge Translation (IKT) lens* [31]	Accept context as inseparable component of a causal chain Use a realist evaluation approach to highlight context in evaluations of IKT	*RQ+ 4 Co-Pro* identifies Contextual Factors as a framework component Use the three Contextual Factors of the framework to categorize and study context-mechanism interactions
*Community-based Participatory Research (CBPR) lens* [32]	Equity is critical to understanding CBPR process & impact for communities, and thus, should be in the foreground of evaluations and informed by various methods and instruments	*RQ+ 4 Co-Pro* names Sub-Dimensions that prioritize and critically interrogate equity: 2.1. Inclusion of Local Knowledge & Ways of Knowing; 2.2. Trust, Power & Mutually Beneficial Partnerships; 2.3. Intersectionality 2.4. Negative Consequences
*Co-Production lens* [33]	Recognize social and transformational effects of co-production, including both those that occur as a part of the research process (as a result of productive interactions) and those related to research results	RQ+ 4 Co-Pro sheds light on the process of engagement and utility of results, by naming both elements in specific Quality Dimensions of the framework (2 & 3), and by doing so, highlights ‘the hidden’ relational and at times transformational benefits of co-production
*Patient & Public Engagement lens* [34]	Increase scientific rigour of framework development	RQ+ 4 Co-Pro is derived from the validity and reliability tested, theory informed, RQ+ approach and framework of the International Development Research Centre
	Include stakeholders in framework development	RQ+ 4 Co-Pro will be field tested in the study described herein, which is structured as a stakeholder inclusive IKT effort.
	Improve accessibility of frameworks (understandability/readability)	Ensure simple, clear, accessible publications in various formats and a well-developed sharing strategy. This publication is one component. As results of this field test emerge, sharing and use strategies will be developed with/by the KUs on the study team.
*Patient & Public Involvement lens* [13]	A single, one-sized fits all framework is unlikely to emerge. Instead, co-develop frameworks for local contexts, principles, and objectives.	RQ+ 4 Co-Pro is a modular construct, whereby new users can adapt and re-shape the framework components to match their values and objectives, while keeping intact the three tenets that address the shortcomings of status quo deliberative, analytic, and RIA evaluation approaches when applied to co-production.
*Public Involvement lens* [26]	Acknowledge: (1) the different rationales for public involvement, (2) that there may be negative impacts, (3) the role of power relations	RQ+ 4 Co-Pro has specific Sub-Dimensions (2.1, 2.2, 2.3, 2.4) that prioritize and will help the field to learn about power dynamics and potentially negative consequences of co-production.

## Study design

This field test will use a multiple method qualitative design. It will include training of participants, standardized data collection using desk-based templates, and follow-up qualitative interviews with both the assessors and those whose projects have been assessed. As well, it will include a consultative process with the project team for revising RQ+ 4 Co-Pro based on the outcomes of the field test [35, 36].

The study will take a research co-production approach. To do so, the study is being undertaken as a partnership between researchers and knowledge users. All activities and responsibilities will be shared, yet, five team members (authors: RKDM, FC, IDG, AK, CM) are primarily responsible for study design and execution. Thirteen team members (authors: ABA, RA, JB, CEC, OD, EDR, LAF, MG, AMH, RK, SK, JR, GS) hold primary responsibility for identifying knowledge uptake and use opportunities. These “knowledge user” team members represent critical stewardship roles for research co-production broadly, including funders, university administrators/leaders, research evaluation specialists, journal editors, co-production trainees, research managers, and co-production scientists. By working together to field test RQ+ 4 Co-Pro, we hope to spark reasoned and appropriate uptake of the framework into settings where current co-production evaluation techniques demand revision and innovation.

**Fig. 3:**
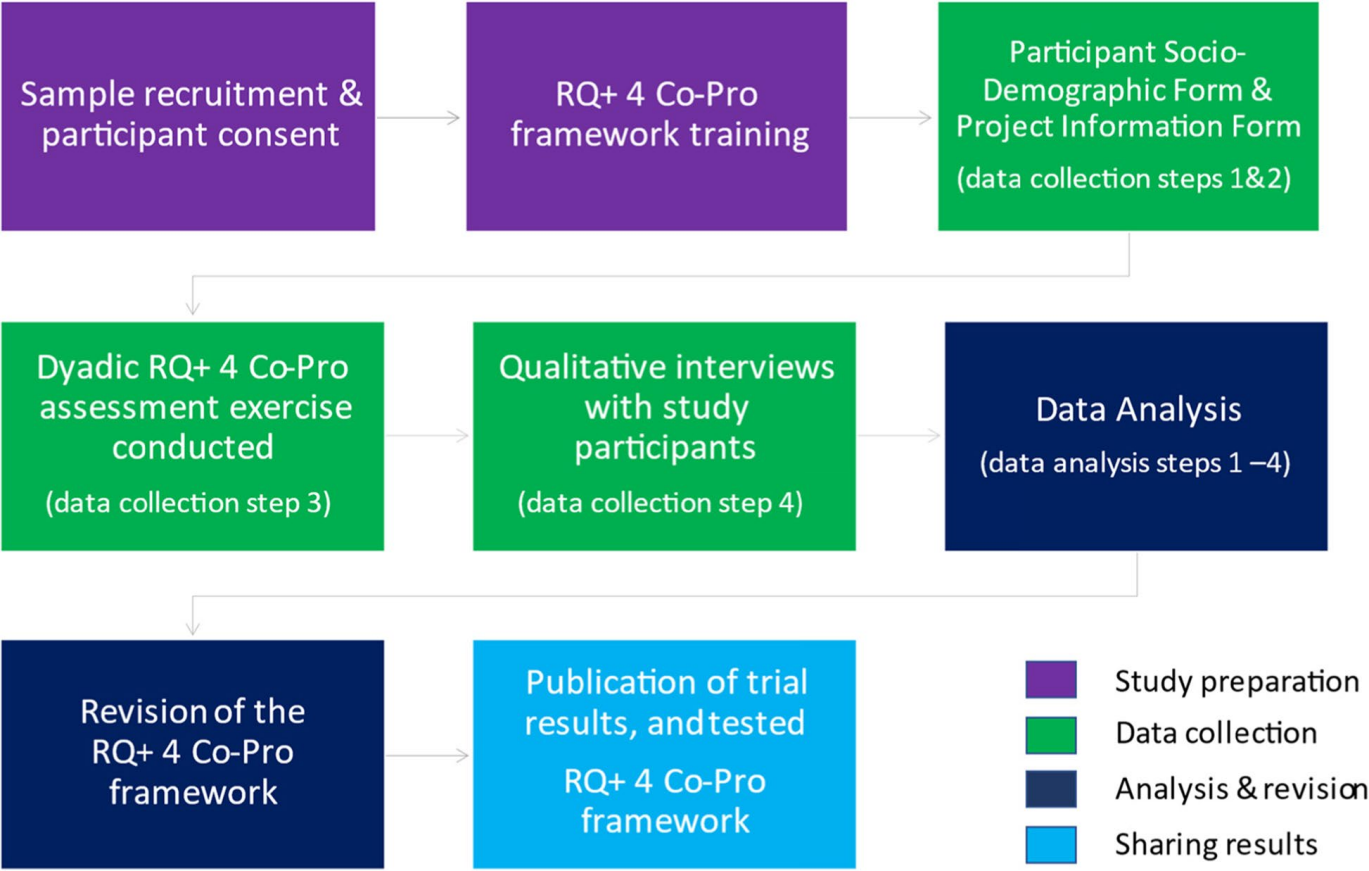
Outline of the research life cycle

The field test will be implemented in four phases, which comprise eight steps. Figure 3 presents an illustration of the complete research life cycle.

### Phase 1—Study preparation

#### Sample recruitment and participant consent

Researchers in the Integrated Knowledge Translation Research Network (IKTRN) will be invited to submit projects for assessment and volunteer to assess another project. IKTRN is a network of researchers with an interest in both using and carrying out research on integrated knowledge translation. IKTRN is funded by a multi-year grant from the Canadian Institutes of Health Research [19]. The sample will be a convenience sample. This sample will be drawn from researchers with IKT research experience (members of the IKTRN) and who have recently completed an IKT project (have an IKT case published in the IKTRN casebook series). This invitation will be delivered by email from the study PI to eligible members of the IKTRN, until the desired sample size of 16 projects is reached, with a maximum of 20 projects. Enrolled participants will be arranged in dyads based on research topic familiarity for the assessment.

The sample range (16–20 projects) is based on two factors. The first is viability given resource requirements of past experiences using the RQ+ approach, and our own study timelines and resources for this project. The second is the anticipated saturation point of qualitative data collected in the field test [37].

##### Eligibility criteria

We will consider eligibility at two levels: (1) the IKT research project and (2) the individuals participating in our study, as described below in Tables 4 and 5 respectively. The research team will gather informed written consent from all participants.

**Table 4:** Project eligibility criteria

Inclusion criteria	Exclusion criteria
✔The research project uses Integrated Knowledge Translation. ✔The project is led by a member of the IKT Research Network ✔The project is either complete or near completion (i.e., has draft products) ✔The project prioritizes health system actors as knowledge users	x Projects focused on science of IKT x Projects focused on training and curriculum development for IKT

**Table 5:** Participant eligibility criteria

Inclusion criteria	Exclusion criteria
✔Individual must have been a member of the IKT Research Network ✔Individual must be a member of the IKT Research Network ✔Individual willing to both submit a project for assessment and act as an assessor in a dyad with another project	x Individual not able to participate in English

#### RQ+ 4 Co-Pro framework training

Participants will receive training in RQ+ 4 Co-Pro. Training will be provided by the core research team, with the aim to introduce the RQ+ 4 Co-Pro framework, the definitions and meaning of its components (contextual factors, quality dimensions and sub-dimensions, evaluative rubrics), and systematize the approach to its use by participants. The 2-h training will be completed prior to the initiation of all data collection.

### Phase 2—Data collection

Data collection will involve four steps: (1) completion of a participant socio-demographic form, (2) completion of a project information form, (3) dyadic RQ+ 4 Co-Pro assessments, and (4) participating in an interview with the research team on the strengths and limitations of the RQ+ 4 Co-Pro framework.

#### Step 1—Participant socio-demographic form

All participants will be sent a link to an online socio-demographic form. This form will collect information on participant demographics and their experience/background developing and/or delivering IKT research projects. We will ask that participants complete this form prior to taking part in the training session (5 min).

#### Step 2—Project information form

All participants will be sent a link to a project information form. This form will be pre-populated by the study team as much as possible to profile the project included in the field test. The study participant will verify and complete any missing information on the project profile prepared by the study team (10 min).

#### Step 3—Dyadic project assessment exercise

Each participant in the paired dyad will provide the other, who will serve as assessor, with publicly available documentation on the project they will be assessing (*inter alia* publications, manuscripts, reports, briefs, blogs, etc). These assessors (study participants in dyads [38]) will review this material to gain an understanding of the project context, as well as its strengths and weaknesses (1-2 hours). Next, the assessors within each dyad will engage in an assessment interview about their projects using the RQ+ 4 Co-Pro training and the field test template (see Annex 1) provided by the research team. Assessment interviews may be done in one virtual call or split in two as the two determine. It is estimated they will last 60 min per project. The field test template will be used by the assessors for recording results of the assessment during the interview.

#### Step 4—Research interview with RQ+ 4 Co-Pro study team

On completion of the dyadic assessments, members of the research team (RKDM, FC) will interview the assessors (study participants) individually using a semi-structured interview guide to elicit their views as both assessor and assessed on the utility and relevance of using RQ+ 4 Co-Pro to assess the quality of IKT research [36]. The interviews will be completed by phone or video conference, depending on participant preference. Interviews will take approximately 60 min.

### Phase 3—Analysis and revision

#### Data analysis

Data analysis will be conducted for each data source independently (Participant demographic forms, Project information forms, Interviews with study participants), and triangulation will be conducted across the independent lines of evidence for congruence as well as instances of discordance.

##### Step 1—Participant demographic form analysis

Frequencies will be generated for all closed-ended questions. Responses to open-ended questions will be analyzed for common and disparate themes using content analysis. Analysis will provide an overview of participants’ backgrounds and experiences brought to the field test.

##### Step 2—Project information form analysis

The project profile forms will be analyzed using content analysis to provide an overview of the nature of the projects included in the field test.

##### Step 3—Interview analysis

Qualitative interview data collection and analysis will occur simultaneously so that identified themes can be incorporated into future interviews. Interviews will be audio recorded with permission of the interviewee. Where permission for transcription or recording are not granted, the interview notes will be sent to the interviewee for review.

We will use thematic analysis [39] to identify patterns in the interview data. We will use an inductive or data-driven approach, without using a pre-existing coding frame. The coding will be modified based on new findings and in collaboration among interviewers. The first two interviews will be coded by two researchers independently and the results compared. Differences will be discussed to ensure agreement on a common approach for the remaining interviews. If agreement is not achieved between the two researchers, a third researcher will arbitrate opposing views and provide a third opinion to reach majority decision if consensus is not achieved.

##### Step 4—Triangulation and analysis

As a final step in data analysis, we will look for similarities and differences of note in the study data by comparing findings across the lines of evidence. We will conduct triangulation by data source and by data collection method. Data will be considered in triangulation by using identified codes and themes to compare data. For example, we may cross tabulate all projects with a timeline of more than 4 years (as identified in the project information form), by perspectives around the importance, or lack thereof, of using contextual factors in the RQ+ 4 Co-Pro framework. This is a hypothetical example. Triangulation will be driven by identified themes in the data.

#### Revision of the RQ+ 4 Co-Pro framework

Based on the findings of the research, we will revise the prototype version of the RQ+ 4 Co-Pro framework. To facilitate this revision, the research team will host a meeting of all team members (including our knowledge users) to review and discuss preliminary research results and how these may induce the desire for change to the framework or its components. The reasons for changing a framework component will relate to the two research questions driving the study: relevance of the framework components and utility of the framework components and its application. Following team iteration, we will prepare any required revisions and represent the revised framework to the study participants for review/member checking.

### Phase 4—Results sharing

The final version of the RQ+ 4 Co-Pro framework will be published in a study findings report. This report will be submitted to an open access peer-reviewed journal for external assessment by co-production specialists. Uptake and use strategies will be developed by/among knowledge user perspectives represented on our co-production team.

## Discussion

This study will yield knowledge about a new way to assess research co-production. Specifically, it will address the relevance and utility of using RQ+ 4 Co-Pro, a framework that includes context as an inseparable component of research, identifies dimensions of quality matched to the aims of co-production, and applies a systematic and transferable evaluative method for reaching conclusions. As we have argued in this paper (see Table 2), evaluation is a needed area of innovation for research co-production to reach its full potential. As we have presented (see Table 3), we are not alone in raising this call.

### Limitations

There are limitations with our study design and study methods. The first is while we propose RQ+ 4 Co-Pro should apply to all research co-production approaches, we have limited our sample to IKT projects as a specific sub-domain of co-production which may limit generalization to other partnered research approaches. Second, our sample of IKTRN projects will prioritize experiences of the global North, as IKTRN membership is largely comprised of members from Canada, Australia, and the UK. Third, we further limited our sample to completed projects and so the study will not test the potential use of RQ+ 4 Co-Pro at the design and implementation stages of co-production projects. Fourth, given the evaluands in our field test are research projects, generalizability to other evaluands such as organizations, project portfolios, or grant applications should be tempered. Fifth, the approach to applying the framework will focus on discussion with principal investigators and documentary analysis. In future uses of the framework, users may wish to be more holistic and include more data sources, for example interviews with end users (however, if the framework is not considered useful to researchers, it will likely be problematic for knowledge users.) At the same time, other applications may go into less depth than we do in this field test, for example using a checklist for project design or application review. Using our design, we cannot be certain our field test experience will generalize to these other potential uses of the framework. Sixth, this field test is limited to health research projects although we recognize that co-production is an approach used in multiple domains of science. Finally, all study participants are part of the same network; this may risk a more positive assessment of each other’s projects due to social bias. However, the goal of the research is to assess the relevance and utility of the framework, not to draw a final conclusion about the quality of co-production research endeavors sampled.

### Looking ahead

We expect that our findings will add to the existing options for assessing co-production research that may benefit researchers but also other stewards of research co-production. Accordingly, we undertake this study as a co-production team with varied experiences and constituents we currently represent. Some potential uses may include funders interested in new ways to select, encourage, and/or evaluate co-production, including at different phases of the research life cycle. It may also give journal editors a higher level of comfort with the quality of research co-production they publish. Research institutions, such as universities or think tanks, may benefit from assessing the quality of co-production they do using a framework tailored to their values, objectives, and context. In the final research report of this RQ+ 4 Co-Pro field test, a section discussing users and uses of RQ+ 4 Co-Pro will be elaborated. We will also tailor outputs and use strategies to the identified needs of knowledge users within our team. These efforts may not appear in peer-reviewed journals or other scholarly publication formats, but instead as use-oriented outputs and activities.

## Supplementary Information

**Additional file 1.**

**Additional file 2.**

## Data Availability

No study data has been collected yet. Upon study completion, please contact the corresponding author for more information.

## Footnotes

1 Applied and use-oriented research refers to any research, however conducted, that focuses on a particular societal issue. Research co-production is also concerned with societal issues but includes the active engagement of the users in the research process itself, from design to completion to implementation of the findings.

2 In our view this should hold for all four pillars of health research: biomedical, clinical, health systems/services, and population and public health.
