# Supplementary material for: Evaluating research co-production: protocol for the Research Quality Plus for Co-Production (RQ+ 4 Co-Pro) framework
Source: Implement Sci Commun. 2022 Mar 14;3:28. doi: 10.1186/s43058-022-00265-7 (PMC8919555; doi:10.1186/s43058-022-00265-7)
Supplement: Supplementary file 1 — Additional file 1. [file 43058_2022_265_MOESM1_ESM.docx]

**RQ+ 4 Co-Pro Field Test Assessment Template**

(Note to journal editors: Language here has been published elsewhere and is maintained for fidelity of the instrument. Published originally in 25.)

**Introduction**

This template provides guidance and the ratings template to test RQ+ 4 Co-Pro. Instructions for setting up the pilot are given separately. The conduct of the pilot will be followed by an interview with the researchers on both your experience carrying out the assessment and your experience of having your project assessed. The goal is to determine the relevance, reliability and value of this repurposing of RQ+ as developed originally by the International Development Research Centre.

Below, we provide definitions of the elements of the assessment (context, dimensions and sub-dimensions), as well as indication of the meaning of scoring values. We cannot in this definition process cover every eventuality as each project and context is unique. We therefore keep the definitions broad on the scoring sheet, you will note that a space for comment is provided at each juncture so that you may include reflections on why you gave the score you gave. Recording this short qualitative justification for your quantitative rubric rating is a required part of the process.

The definitions provided here were first developed in a chapter for a book on Research Co-Production (McLean, Graham & Carden, forthcoming). Definitions of scoring in the rubric have been added.

In the descriptions that follow, the language outlines an application at the end of a research activity. This is consistent with the sample that will be addressed in the IKTRN trial. In future applications, RQ+ 4 Co-Pro may be adapted to facilitate co-production design or management.

**Contextual Factors**

Research always occurs in political, economic, sociological, and natural settings. This is particularly true in the case of co-production. RQ+ 4 Co-Pro identifies three contextual factors that can be monitored and categorized in a co-production project or program evaluation. By studying these factors, users of *RQ+ 4 Co-Pro* can learn, share experience, and cultivate enabling environments for co-production work. In the more immediate term, categorizing research context can help project coordinators, funders, or managers understand risk factors and identify mitigation strategies for individual projects or for monitoring project portfolios. Classifications of context are done independently of ratings against the quality dimensions, and they are not intended to modify project quality ratings. The intent is to document and understand the project environment. One categorization is not meant to imply ‘better than’ another categorization. The three *RQ+ 4 Co-Pro* contextual factors are: 1) Knowledge Use Environment; 2) Research Environment; 3) Capacities for Co-Production.

1. Knowledge Use Environment

This contextual factor addresses the absorptive capacity of the knowledge use environment. This typically stems from the broad environment and culture of the knowledge-user partner and then manifests for the co-production team. The knowledge use environment may be highly empowering, with a strong appetite for research and evidence to inform policy, program, practice or product improvement. Here, resources and incentives will encourage and reward the use of evidence in decision-making. Alternatively, the environment may be restrictive; as a result, the co-production team faces significant barriers, even professional risks, to research evidence vis-à-vis alternative decision-making approaches. In a restrictive environment, resources and incentives do not support research uptake and use.

| 1. Restrictive | 1. Unsupportive | 1. Supportive | 1. Empowering |
| --- | --- | --- | --- |
| There is disinterest or opposition to the role of research. Research processes and/or products are overlooked or challenged. | While there may be a claim of the importance of using evidence, there is limited evidence that research is valued in decision-making processes. There may be a lack of awareness as to the role or utility of research. | There is support for the importance of research and evidence, and an awareness of how research might improve decision-making. Research is generally valued and does not face avoidance and/or skepticism. | There is clear demand for research and evidence. There is active encouragement for research, a thorough understanding of its requirements, and a demonstrable intent to use evidence to inform decisions and directions. |
| Rating: |  |  |  |
| Comments & Justification | | | |

2. Research Environment

This contextual factor addresses the environment in which the researcher partner(s) in the co-production team works. In some circumstances the environment may empower co-production as a valid means of knowledge generation and provide researchers incentives, resources, and rewards for good practice. Alternatively, co-production may be an undervalued or diminished means of conducting science where researchers may be explicitly or implicitly discouraged from undertaking co-production and thus put career progression and peer acceptance at risk by engaging knowledge users in their research.

| 1. Restrictive | 1. Unsupportive | 1. Supportive | 1. Empowering |
| --- | --- | --- | --- |
| There is disinterest or opposition to co-production research. Co-production processes and/or products are dismissed for low credibility, and this may negatively influence perceptions of those undertaking co-production vis-a-vis their peers. This may be explicit or implicit. | While there are no explicit negative assumptions about co-production, there is limited evidence that co-production is valued similarly to traditional curiosity-driven science. There may be instances of implicit bias against co-production work, and a lack of awareness as to the specificities of co-production from peers and institutional leadership. | There is support in the research environment for co-production work. Co-production results are valued equally to curiosity-driven science. The process is encouraged and supported by peers. Co-producers do not face academic setbacks. | Co-production research is incentivised, resourced and valued. Co-producers' results and processes are valued equally to other forms of science, and when done well, encouraged as a legitimate and rigorous means of knowledge generation. Co-producers receive specialized academic incentives and rewards for good work. |
| Rating: |  |  |  |
| Comments & Justification: | | | |

3. Capacities for Co-Production

This factor categorizes the extent to which the research places focus on training and developing co-production practice and/or theory amongst researchers and knowledge users. We track this contextual factor because co-production is a young and emerging field, and nurturing the next generation is required for future acceptance and sustainability. When the focus is strong, a considerable amount of time and resources are devoted to purposefully and consciously developing the skills of team members and aptitude for co-production is envisaged as a positive result of the effort (amongst both researchers and knowledge users). Alternatively, capacity building in co-production may not be a deliberate part of the research effort. This is identified when no discernable resources are devoted to it, and the only viable skill development opportunity for researchers or knowledge users will come from learning by doing. This may be the case with a highly experienced or beginner co-production team. Unlike the other contextual factors, Capacities for Co-Production does not denote a measure of risk. This is not an outcome measure. It is a measure of the intensity of the effort.

| 1. No Focus | 1. Minimal Focus | 1. Significant Focus | 1. Strong Focus |
| --- | --- | --- | --- |
| The team has no access to support (training, mentors, resources, etc.) to develop co-production skills. No documentation indicates co-production capacity strengthening is a goal or objective of the work. It is possible capacity is/was built, but this was clearly an unintentional outcome of the project. | The team may receive general capacity building, but little if any, addresses co-production specific skills. Through ‘learning by doing’ some capacity strengthening is likely to occur. Capacity strengthening was a goal, but co-production specific skills were not prioritized. | There is evidence to indicate a capacity strengthening plan was developed for/by the project, and it has been enacted. The team is learning by doing and through training. There are demonstrable capacity strengthening outcomes expected. | There is a clear co-production learning plan built into the project and meaningfully enacted. The team places strong focus on strengthening co-production capacities specifically through the process, and capacity strengthening is envisioned as an important objective and outcome of the project. |
| Rating: |  |  |  |
| Comments & Justification: | | | |

**Quality Dimensions and Sub-Dimensions**

Any judgement of research quality should reflect the values underpinning that research effort. RQ+ 4 Co-Pro articulates three dimensions, and eight corresponding sub-dimensions of quality that reflect broad values for partnered research.

Co-production research must be scientifically robust, thus we begin with ‘Scientific Rigour’, a non-negotiable component of any co-production effort. The second dimension of *RQ+ 4 Co-Pro*, Research Legitimacy, highlights four sub-dimensions that together measure the fidelity of the research effort to the environment in which it occurs and the results it will produce for intended beneficiaries. The third dimension, Positioning for Use, examines the relevance of the research to the needs of users and the openness and actionability of the process and results.

In the *RQ+ 4 Co-Pro* Framework, these dimensions are not independent variables, they are interrelated. Yet, by disaggregating and allowing focus on each component, *RQ+ 4 Co-Pro* highlights and brings importance to the alternative and diverse qualities that ultimately underpin excellence in co-production. For our purposes the dimensions hold equal weight. Other users may choose to weigh dimensions differently in order to increase focus on challenging or significant components of their work. The eight-point rubric, is as follows:

| Insufficient data to assess | Unacceptable | | Less than acceptable | | Good | | Very good | |
| --- | --- | --- | --- | --- | --- | --- | --- | --- |
| IIA | 1 | 2 | 3 | 4 | 5 | 6 | 7 | 8 |

**1.Scientific Rigour**

The first dimension of research co-production quality addresses the technical merit and demonstrable excellence of the research. This requires an examination of the project vis-à-vis the standards and expectations of the methodological approach (qualitative research, clinical trials, statistical methods, ethnographic immersion, for example). Meaningful co-production partnerships are considered across any and all fields. Yet, this part of the assessment must be considered vis-à-vis the intentions of the work and the fair expectations of the knowledge-user partners. In some circumstances engagement will be necessary from start to finish, in other cases knowledge users and researchers will have negotiated mutually beneficial terms, and these idiosyncrasies should be considered and examined here. The dimension is represented with two distinct sub-dimensions.

- 1. Protocol

This measure of quality addresses the design of the research project using the accepted best practices of the field. It examines how the study is framed in the current knowledge, reproducibility of the design, how methodological standards are met or exceeded with viable innovations, and the overall design openness. This dimension also considers the co-production process, including when and how engagement is built in.

| Insufficient data to assess | Unacceptable | | Less than acceptable | | Good | | Very Good | |
| --- | --- | --- | --- | --- | --- | --- | --- | --- |
| IDA | 1 | 2 | 3 | 4 | 5 | 6 | 7 | 8 |
|  | The protocol is not acceptable to meet the standard of the field. It may represent a wasteful (duplicative, unusable) study. | | The protocol should have been revised to address important gaps or weaknesses | | The protocol is acceptable. Well grounded in the current knowledge base, and raises important questions and methods to answer them. | | The protocol is clear, thorough and transparent. It represents a sophisticated and innovative design in its domain. | |
| Rating |  |  |  |  |  |  |  |  |
| Comments & Justification: | | | | | | | | |

- 1. Methodological Integrity

Refers to the technical fidelity of protocol implementation and research management decisions. This will include how principles of working practice are established and navigated by the full co-production team. How partnerships are managed is essential throughout each part of the process, which will typically examine issues such as: (i) research questions are pursued rigorously (ii) adequate and appropriate data collection is conducted, (iii) relevant analysis frameworks are selected and applied according to best practice and knowledge-user needs, (iv) conclusions are grounded in data collected, and, (v) clear and accurate presentation of results in light of knowledge-user contexts and needs.

| Insufficient Data to Assess | Unacceptable | | Less than acceptable | | Good | | Very Good | |
| --- | --- | --- | --- | --- | --- | --- | --- | --- |
| IDA | 1 | 2 | 3 | 4 | 5 | 6 | 7 | 8 |
|  | The protocol was not followed, and no discernable or reasonable justification was provided. | | Significant changes to the research were made outside the protocol with limited rationale. | | The research implementation generally followed the protocol and most adaptations were reasonable and could be justified. | | The research implementation followed the protocol very well, clearly rationalizing any adaptations where required by new knowledge of, or changes in, context or emerging issues in the coproduction process. | |
| Rating |  |  |  |  |  |  |  |  |
| Comments & Justification: | | | | | | | | |

**2. Research Legitimacy**

Legitimacy addresses the fidelity of the research to the context in which it is or will be implemented. In the context of co-production, legitimacy includes sub-dimensions related to fairness and meaning in knowledge generation, diversity, equity and inclusion, and meaningful relationships being created and/or sustained between all partners involved in the co-production effort. Specifically, Research Legitimacy is represented in four sub-dimensions.

2.1 Inclusion of Local Knowledge and Ways of Knowing

This sub-dimension addresses the degree to which the research is grounded in the reality and knowledge base of the intended users and beneficiaries of the work. Exemplary projects will ensure scientific methods embrace and empower the realities of local ways of knowing, existing cultures, and norms or expectations about knowledge. These could be cultural, commercial, organizational, or political knowledge localities, depending on the aims and context of the project. Attention must be paid to decolonizing local standards from predominant scientific standards surrounding knowledge and evidence, and appropriately weighing all partners’ perspectives.

| Insufficient Data to Assess | Unacceptable | | Less than acceptable | | Good | | Very Good | |
| --- | --- | --- | --- | --- | --- | --- | --- | --- |
| IDA | 1 | 2 | 3 | 4 | 5 | 6 | 7 | 8 |
|  | The research ignores local realities and knowledge. It implements a methodology driven by external interests and experience. | | The research is insufficiently grounded in local realities and knowledge and significant gaps remain. | | The research is sufficiently grounded in local realities and integrates local and professional knowledge with some inconsistencies. | | The research is fully grounded in local realities and context, integrating relevant local and professional knowledge and embraces ways of knowing relevant to the users and beneficiaries. | |
| Rating |  |  |  |  |  |  |  |  |
| Comments & Justification: | | | | | | | | |

2.2 Trust, Power, and Mutually Beneficial Partnership

This sub-dimension examines the underlying power dynamics of the research process, specifically examining how power was created, shared, and sustained. It also interrogates if/how the co-production effort is designed and managed to address the needs and desires of all parties throughout the research process. A mutually beneficial partnership does not mean all tasks and resources are shared equally; it means decisions about how tasks and resources are utilized are mutually endorsed.

| Insufficient Data to Assess | Unacceptable | | Less than acceptable | | Good | | Very Good | |
| --- | --- | --- | --- | --- | --- | --- | --- | --- |
| IDA | 1 | 2 | 3 | 4 | 5 | 6 | 7 | 8 |
|  | There is evidence of a significant power imbalance in the research relationship. | | There is some evidence that efforts were made to negotiate the partnership but significant problems were encountered in implementation, suggesting inadequate negotiation. | | There is clear evidence the partnership was negotiated and freely agreed by both researchers and knowledge users and was largely sustained in the research process with some challenges. | | There is clear evidence that the partnership was negotiated and freely agreed by both researchers and knowledge users. It has been sustained throughout the research process. | |
| Rating |  |  |  |  |  |  |  |  |
| Comments & Justification: | | | | | | | | |

2.3. Intersectionality

This sub-dimension addresses the degree to which the research takes account of the varied perspectives underpinning the work and produces equitable processes and outcomes for different intersectional connections with the work. Issues of diversity, equity, and inclusion are considered here. Very good research will be sensitive to the social environment in which the research takes place and cognizant of the potential biases the co-production team brings to the work. Intersectionality is a critical element in each of the design, conduct and implementation components of the work. The assessment should focus on the extent to which intersectionality is considered and built into each phase of the project. In the case of an impact assessment, it may examine outcomes for varied intersectional groups. In the case of a needs assessment, it may examine whose needs are being considered and whose are not, or how they are being valued and why. No co-production project should be blind to intersectional considerations.

| Insufficient Data to Assess | Unacceptable | | Less than acceptable | | Good | | Very Good | |
| --- | --- | --- | --- | --- | --- | --- | --- | --- |
| IDA | 1 | 2 | 3 | 4 | 5 | 6 | 7 | 8 |
|  | The research does not address intersectionality sufficiently to be acceptable. Generally, it is blind to issues of gender, culture, and other intersections which should be pertinent to the work. | | Although attempts were made, the research is weak in its consideration of intersectionality issues and/or is weak adapting to new knowledge that emerges. | | The research takes adequate account of relevant intersectionality issues in analysis and reporting and is able to adapt in most cases to new issues that emerge. | | The research takes account of all relevant intersectionality issues and adapts as new learning about these is taken on by the team. The research goes well beyond dis-aggregation of data in analysis or reporting, and makes intersectional realizations are strong component of the choice of questions, methods, analytical frameworks and reporting/sharing approaches. | |
| Rating |  |  |  |  |  |  |  |  |
| Comments & Justification: | | | | | | | | |

2.4. Attention to Potentially Negative Consequences

This sub-dimension refers to the strategies employed in the co-production project to minimize and mitigate any negative consequences of the work, whether expected or unexpected. Negative consequences could include damages to individual partners or their organizations, damages to participants, adverse outcomes for beneficiary communities, or damages to the natural environment. Evidence of exemplary performance is found in ethics adherence through the research co-production, but also in the way user/beneficiary relationships are managed and how these perspectives are valued in how decisions about project progress are made.

| Insufficient Data to Assess | Unacceptable | | Less than acceptable | | Good | | Very Good | |
| --- | --- | --- | --- | --- | --- | --- | --- | --- |
| IDA | 1 | 2 | 3 | 4 | 5 | 6 | 7 | 8 |
|  | The research is poor in adherence to the research protocol and does not track for negative effects. | | The research does not adhere sufficiently to the research protocol and has poor systems for tracking negative effects during implementation. | | The research adheres to an ethics protocol and has included measures to monitor and mitigate negative effects or outcomes. | | The research adheres to a strong ethics protocol and is vigilant about and responsive in identifying potentially negative effects resulting from the research for participants or impacted communities. Clear evidence indicates how these protocols were developed and used. | |
| Rating |  |  |  |  |  |  |  |  |
| Comments & Justification: | | | | | | | | |

**3.Positioning for Use**

Positioning for Use addresses the extent to which the co-production process enhanced the likelihood of research uptake and impact. A first critical element is how relevant the research objectives and questions are for the intended beneficiaries and/or users of the work. Second is the creation of audience-friendly and open access research outputs and results. User engagement as a means of facilitating knowledge translation is a matter of scientific rigour in co-production: thus, it is assessed specifically under quality dimension one.

3.1. Relevance

This sub-dimension reflects the extent to which the research takes on existing and predominant societal or practical problems of relevance to knowledge users. The measure examines how the research was prioritized, who it serves, and how widely endorsed the needs and challenges it addresses are by co-producers and impacted organizations or communities.

| Insufficient Data to Assess | Unacceptable | | Less than acceptable | | Good | | Very Good | |
| --- | --- | --- | --- | --- | --- | --- | --- | --- |
| IDA | 1 | 2 | 3 | 4 | 5 | 6 | 7 | 8 |
|  | The research has no relevance to the local community/user. | | The research topic and approach is only marginally relevant and should be revisited with knowledge users. | | The research topic is relevant to knowledge users and serves a community need. (ie maybe not the highest need in that community.) | | The research topic is highly relevant to knowledge users, and is clearly intended to serve their and their communities’ needs. | |
| Rating |  |  |  |  |  |  |  |  |
| Comments & Justification: | | | | | | | | |

3.2. Openness & Actionability

This sub-dimension addresses how research is conducted and how results are tailored into outputs, products, and results that are useful, attractive, and understandable for knowledge users. The useability of the solution generated is considered, and so is the presentation of the solution in an engaging format. This includes how openly available (open access), applicable, tailored, and timely the conduct and results are for action.

| Insufficient Data to Assess | Unacceptable | | Less than acceptable | | Good | | Very Good | |
| --- | --- | --- | --- | --- | --- | --- | --- | --- |
| IDA | 1 | 2 | 3 | 4 | 5 | 6 | 7 | 8 |
|  | The presentation of findings does not address issues of accessibility, and the needs of users. Perhaps only published and shared in academic journals or not at all. | | The presentation of the findings is limited and not meaningful for all intended users. The majority of sharing efforts have been placed on academic priorities. | | The findings are available, clear and presented in ways and language that are usable by all intended users. | | The findings are available, clear, and presented in ways and language that are usable by all intended users. The research included sophisticated stakeholder mapping to capture all relevant users and tailored sharing strategies for each identified group through the research process. | |
| Rating |  |  |  |  |  |  |  |  |
| Comments & Justification: | | | | | | | | |
