# Supplementary material for: Evaluating research co-production: protocol for the Research Quality Plus for Co-Production (RQ+ 4 Co-Pro) framework
Source: Implement Sci Commun. 2022 Mar 14;3:28. doi: 10.1186/s43058-022-00265-7 (PMC8919555; doi:10.1186/s43058-022-00265-7)
Supplement: Supplementary file 2 — Additional file 2. [file 43058_2022_265_MOESM2_ESM.docx]

**Crosswalk of the RQ+ framework with the RQ+ 4 Co-Pro framework**

|  | **RQ+** | **RQ+ 4 Co-Pro** |
| --- | --- | --- |
| **Purpose** | To assess the quality of research for development | To assess the quality of research co-production |
| **Framework Developer** | Funding agency & researchers (International Development Research Centre & its research community) | RQ+ 4 Co-Pro team (researchers, evaluators, university administrators, journal editors, students, funders, not-for-profit health advocates) |
| **Primary Reference** | 29 | 25 |
| **Comparison of Framework Components** | | |
| **Contextual Factors** | 1. Maturity of the Research Field  2. Data Environment  3. Political Environment  4. Organizational Environment  5. Research Capacity Strengthening | - - - 1. Knowledge Use Environment       2. Research Environment       3. Capacities for Co-Production |
| **Quality Dimensions & Sub-Dimensions** | **1. Scientific Rigour**  1.1. Study Protocol  1.2. Methodological Integrity  **2. Research Legitimacy**  2.1. Addressing Potentially Negative Consequences  2.2. Gender  2.3. Inclusiveness  2.4. Engagement with Local Knowledge  **3. Research Importance**  3.1. Originality  3.2. Relevance  **4. Positioning for Use**  4.1. User Engagement  4.2. Openness & Actionability | **1. Scientific Rigour**  1.1. Study Protocol  1.2. Methodological Integrity  **2. Research Legitimacy**  2.1. Inclusion of Local Knowledge and Ways of Knowing  2.2. Trust, Power and Mutually Beneficial Partnership  2.3. Intersectionality  2.4. Attention to Negative Consequences  **3. Positioning for Use**  3.1. Relevance  3.2. Openness and Actionability |
